# Neurofilament Light Chain Is a Biomarker of Neurodegeneration in Ataxia Telangiectasia

**DOI:** 10.1007/s12311-021-01257-4

**Published:** 2021-04-24

**Authors:** H. Donath, S. Woelke, R. Schubert, M. Kieslich, M. Theis, G. Auburger, R. P. Duecker, S. Zielen

**Affiliations:** 1 Division of Allergology, Pulmonology and Cystic Fibrosis, Department for Children and Adolescents, Goethe University, Frankfurt, Germany; 2 Division of Pediatric Neurology, Department for Children and Adolescents, Goethe University, Frankfurt, Germany; 3 Experimental Neurology, Medical School, Goethe University, Frankfurt, Germany

**Keywords:** Ataxia telangiectasia, Neurofilament light chain, NfL, Neurodegeneration, Disease progression, Biomarker

## Abstract

Ataxia telangiectasia (A-T) is a progressive and life-limiting disease associated with cerebellar ataxia due to progressive cerebellar degeneration. In addition to ataxia, which is described in detail, the presence of chorea, dystonia, oculomotor apraxia, athetosis, parkinsonism, and myoclonia are typical manifestations of the disease. The study aimed to evaluate the specificity and sensitivity of neurofilament light chain (NfL) as a biomarker of neurodegeneration in relation to SARA score. In this prospective trial, one visit of 42 A-T patients aged 1.3–25.6 years (mean 11.6 ± 7.3 years) was performed, in which NfL was determined from serum by ELISA. Additionally, a neurological examination of the patients was performed. Blood was collected from 19 healthy volunteers ≥ 12 years of age. We found significantly increased levels of NfL in patients with A-T compared to healthy controls (21.5 ± 3.6 pg/mL vs. 9.3 ± 0.49 pg/mL, *p* ≤ 0.01). There was a significant correlation of NfL with age, AFP, and SARA. NfL is a new potential progression biomarker in blood for neurodegeneration in A-T which increases with age.

## Background

Ataxia telangiectasia (A-T) is a pleiotropic and devastating human autosomal recessive disorder with genetic instability. Prominent features are cerebellar degeneration, oculocutaneous telangiectasia, immunodeficiency, cancer predisposition, and endocrinological abnormalities, such as insulin resistance, diabetes, and growth retardation [1, 2]. Recurrent infections and aspiration contribute to lung disease and favor the development of bronchiectasis and respiratory failure [3, 4].

In recent years, various therapeutic approaches like bone marrow transplantation [5–7], dexamethasone treatment [8–10], and gene therapy [11–13] have been developed to positively influence the course of A-T, but still no treatment to cure the disease is available. There is considerable clinical variation between patients with A-T. The most important prognostic marker is the presence of residual ATM kinase activity which leads to the milder “variant” phenotype, while IgG2 deficiency and the hyper-IgM-phenotype are reverse markers [14–16].

Given the fatality of the disease [17], there is an urgent need to identify tissue- and organ-specific surrogate markers for disease progression. Among all the affected organ systems, neurodegeneration has the greatest influence on quality of life, especially at a young age.

The neuropathological changes in A-T show a macroscopically visible cerebellar atrophy with a thinning of cerebellar hemispheric foliae and cerebellar vermis. Cause for the degeneration of the cerebellar white matter is a loss of Purkinje, granular, and basket cells [18–20]. Imaging studies repeatedly demonstrated cerebellar atrophy in all patients with classical A-T at advanced stages [21, 22]. In contrast, magnetic resonance imaging (MRI) studies without laborious voxel-based volumetry hardly ever revealed pathological findings in younger patients with A-T (23, own data unpublished). Several neurological scales for A-T have been proposed [23, 24]. However, these scales do not clearly identify the patients who are likely to have a poor prognosis and depend on the patient’s cooperation, which can be a crucial problem in a pediatric cohort. In addition, these markers are not sensitive enough to evaluate disease progression once patients are bound to wheelchairs.

Because the diagnosis of A-T is made earlier, in some cases even in the pre-atactic stage, as is the case with abnormal newborn screening [25], there is an urgent need for a surrogate marker of disease progression, particularly for neurodegeneration, as imaging techniques or examination scores only detect damage when it has become widespread and irreversible. While alpha fetoprotein (AFP) is a good diagnostic biomarker for A-T, it is not suitable for assessing disease progression or severity. In contrast, neurofilament light chain (NfL) is a tissue-specific biomarker that is directly related to neurodegeneration and probably useful as a surrogate marker of disease progression. However, it is not disease-specific and cannot replace the value of AFP in diagnosis.

Neurofilaments can be detected and reliably quantified in blood serum as specific biomarkers of neuroaxonal damage. As part of the neuronal cytoskeleton, they are target of constant renewal and degradation. In healthy individuals, there is a permanent release into cerebrospinal fluid (CSF) and serum at low concentrations [26–28]. In the context of acute axoneuronal and synaptic damage, serum concentrations increase explosively within days to weeks regardless of the etiology of injury [28]. It is unclear whether the NfL increase in extracellular fluids is caused passively by damage or actively by remodeling processes after brain tissue damage [29]. For spinocerebellar ataxia type 3 (SCA3), it has recently been shown that serum concentrations of NfL correlate with the severity of ataxia [30].

In the current study, we examined serum concentrations of NfL as an easily accessible, neuron-specific, and objective blood biomarker of neurodegeneration in A-T. We correlated this potential surrogate marker of disease progression to the scale for the assessment and rating of ataxia (SARA) [24] in order to evaluate the clinical significance of alterations. Additionally, NfL was correlated to age, AFP, neuropathy, wheelchair dependency, immunological findings, hepatopathy, diabetes, dystonia, and myoclonus.

## Methods

### Eligibility

Between February and September 2020, 42 A-T patients (aged 1.3–25.6 years) were investigated prospectively with SARA [24]. In addition, NfL and AFP were determined in the serum of whole blood. The results were compared to NfL measures of 19 healthy controls ≥12 years of age. Healthy controls were recruited by public posting. Due to ethical concerns, no children under the age of 12 years were recruited for blood withdrawal. Written consent from patients or caregivers was required for each subject and healthy control. Participation in an ongoing clinical trial, presence of malignant disease, or recent neurosurgery operation/intervention was defined as exclusion criteria.

The A-T patients were genetically and/or clinically diagnosed according to recent World Health Organization (WHO) recommendations [31].

### Data Ascertainment

The data presented were collected from a non-interventional clinical trial at the children’s hospital, Goethe University, Frankfurt. The trial was registered at clinicaltrials.gov 2020. The study was approved by the responsible ethics committee in Frankfurt (application number 168/18). The study was conducted following the ethical principles of the Declaration of Helsinki, regulatory requirements, and the code of Good Clinical Practice.

### NfL-ELISA

Serum samples of 42 A-T patients and 19 healthy controls were collected and frozen at −80 °C. NfL concentration was measured with the highly sensitive enzyme-linked immunosorbent assay kit for neurofilament from Cloud-Clone Corp. (Houston, USA) according to the manufacturer’s protocol. Briefly, 100 μL of serum samples and standards with a biotin-conjugated antibody specific to NfL were added to the NfL antibody–specific pre-coated microplate and incubated for 1 h at 37 °C. Then, 100 μL of detection reagent A was added to each well and incubated for another 1 h at 37 °C. Each well was washed three times with wash buffer before 100 μL of detection reagent B was added to each well, and the plate was incubated for 30 min at 37 °C. After an additional washing step, 90 μL of substrate solution was added to each well and the wells were incubated for 15 min at 37 °C. The enzyme substrate reaction was terminated by adding 50 μL of stop solution to each well and the measurements were conducted at 450 nm immediately.

### Clinical Findings

Peripheral neuropathy was defined as attenuated reflex status and/or pathologic nerve conduction velocity. Hepatopathy was diagnosed by elevated transaminases as well as sonographic tissue changes. Diabetes was assumed to be pathologically elevated HbA1c and/or fasting glucose as well as previously medically diagnosed diabetes.

### Statistical analysis

For statistical analysis, GraphPad Prism 5.01 (GraphPad Software, Inc.) and R version 4.0.3 were used. Values are presented as arithmetic means with standard deviations (SDs). For comparisons between the two study groups, the two-tailed Mann-Whitney *U*-test was applied. Correlations were analyzed by Spearman’s correlation coefficient. *p*-values ≤ 0.05 were considered significant.

## Results

Patients’ characteristics are shown in Table 1. No variant A-T patient was included in the study.

**Table 1:** Demographic and health characteristics of patients

	A-T (*n* = 42)	Healthy controls (*n* = 19)	*p*-value
Age (years)	11.6 ± 7.3	18.6 ± 4.4	≤ 0.01
Sex (f/m)	23/19	9/10	
NfL (pg/mL)	21.5 ± 3.6	9.3 ± 0.49	≤ 0.01
SARA (points)	20.4 ± 8.2	N/A	
IgG substitution	9/42	N/A	
Granuloma	5/42	N/A	
History of cancer	4/42 *n* = 1 meningioma	N/A	
Peripheral neuropathy	11/42	N/A	
Dystonia	3/42	N/A	
Myoclonus	12/42	N/A	
Wheelchair-bound	18/42	N/A	
IgA deficiency	32/42	N/A	
Hepatopathy	12/42	N/A	
Diabetes	4/42	N/A	

Values are shown as mean ± SD. SARA scores were available from 28 patients

Forty-two patients with A-T and 19 healthy controls were included in the study. The mean age in the A-T cohort was 11.6 ± 7.3 years compared to 18.6 ± 4.4 years in the healthy control group.

**Fig. 1:**
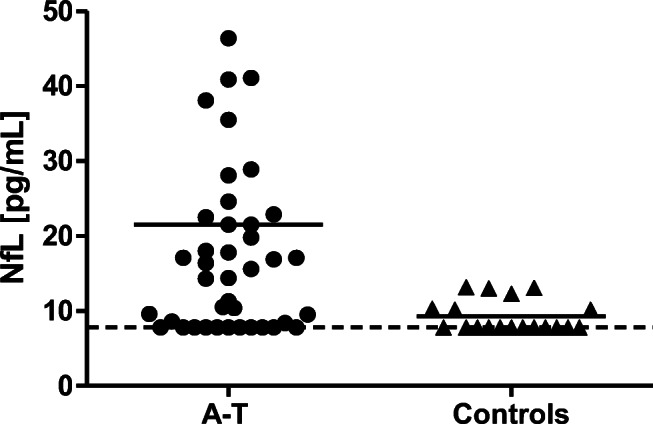
Comparison of NfL serum levels between A-T patients (*n* = 42) and healthy controls (*n* = 19). The hatched line marks the detection limit of 7.8 pg/mL

**Fig. 2:**
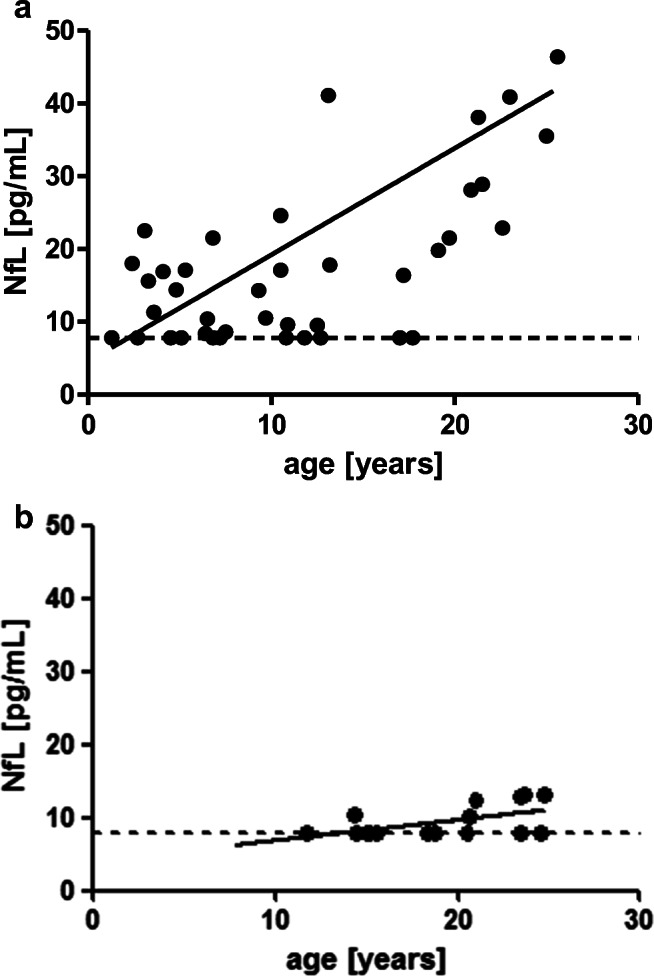
**a** Correlation of NfL with age in A-T patients (*r* = 0.45, *p* ≤ 0.01). The hatched line marks the detection limit of 7.8 pg/mL. **b** Correlation of NfL with age in healthy controls (*r* = 0.58, *p* ≤ 0.01) The hatched line marks the detection limit of 7.8 pg/mL.

We found significantly increased levels of NfL in patients with A-T compared to healthy controls (21.5 ± 3.6 pg/mL vs. 9.3 ± 0.49 pg/mL, *p* ≤ 0.01) (Fig. 1). There was a significant correlation of NfL with age in A-T patients (*r* = 0.45, *p* ≤ 0.01) as shown in Fig. 2a. In healthy controls, we could also show a significant correlation of NfL with age (*r* = 0.58, *p* ≤ 0.01) as shown in Fig. 2b. For the healthy controls, this corresponds to an average increase in NfL of 0.31 pg/mL/year, while for the A-T patients, the average increase is 1.49 pg/mL/year.

**Fig. 3:**
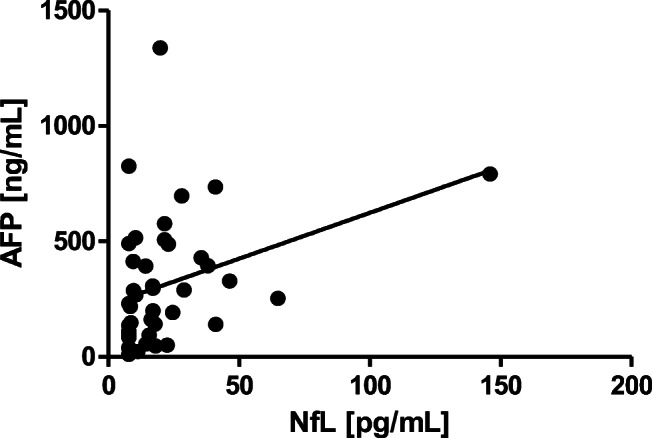
Correlation of AFP with NfL (*r* = 0.34, *p* ≤ 0.05)

In A-T patients, AFP correlated significantly with NfL (*r* = 0.34, *p* ≤ 0.05) (Fig. 3).

**Fig. 4:**
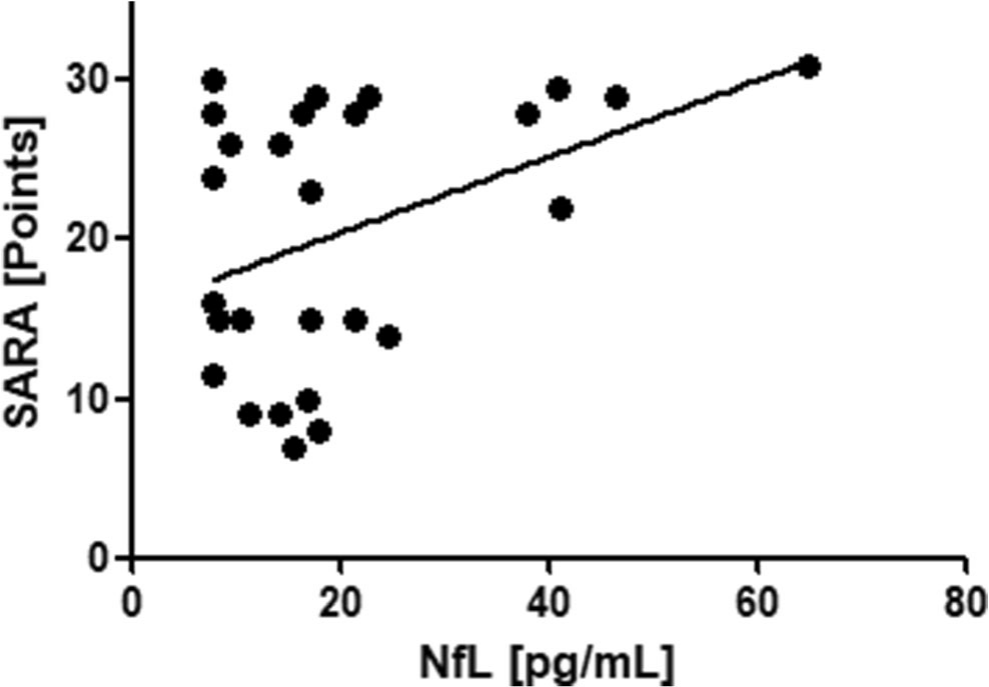
Correlation of NfL with SARA in A-T patients (*r* = 0.41, *p* ≤ 0.05)

The SARA score was available from 28 A-T patients and correlated significantly with NfL (*r* = 0.41, *p* ≤ 0.05) (Fig. 4). We could show a significant correlation of NfL with wheelchair dependency (*r* = 0.37, *p* ≤ 0.05) and peripheral neuropathy (*r* = 0.55, *p* ≤ 0.001). Dystonia did not correlate with NfL (dystonia: *r* = 0.05, *p* > 0.05), while myoclonus did (r = 0.37, *p* ≤ 0.05).

Regarding immunological characteristics, we could not show a correlation for IgA deficiency (*r* = –0.11, *p* > 0.05) and granulomas (*r* = -0.06, *p* > 0.05). Concerning metabolic risk factors, diabetes (*r* = 0.39, *p* < 0.05) and hepatopathy (*r* = 0.51, *p* ≤ 0.001) correlated with NfL, suggesting that diabetes, hepatopathy, neuropathy, and NfL show prominent parallel progression with age.

**Fig. 5:**
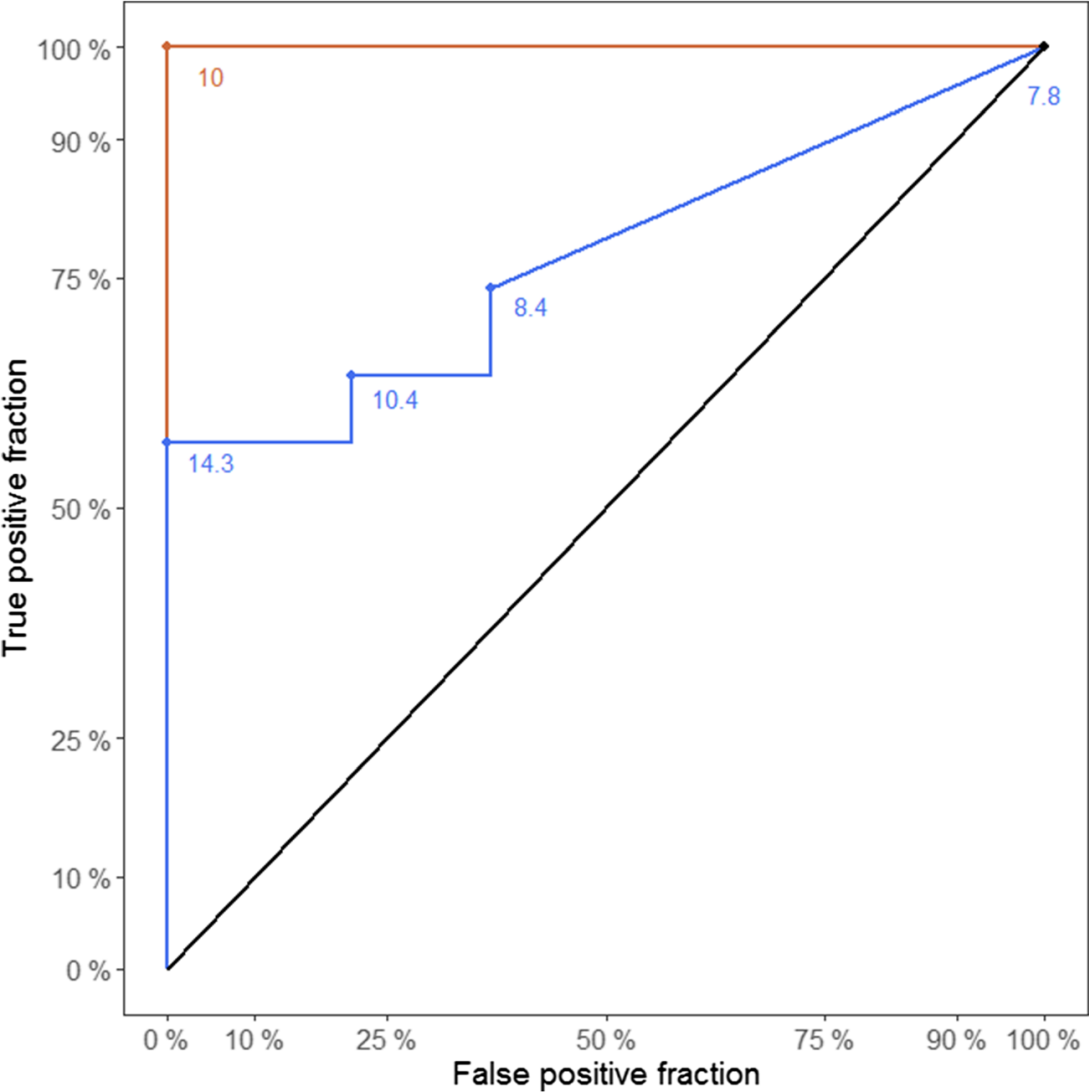
ROC analysis of NfL (blue) and AFP (red)

Figure 5 shows a receiver operating characteristic (ROC) analysis to discriminate between the sensitivity and specificity of AFP and NfL.

**Fig. 6:**
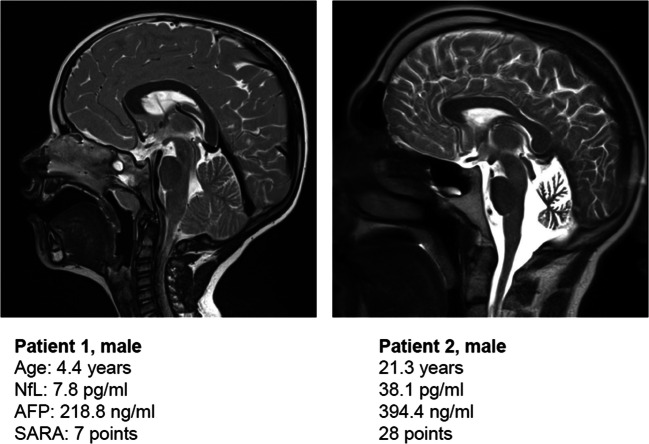
MRI scans (T2) of two A-T patients. Patient 1: a 4.4-year-old boy with mild neurological deficit, SARA of 7 points, and normal NfL. Patient 2: a 21.3-year-old man with severe neurological deficit, SARA of 28 points, and high levels of NfL

Figure 6 shows exemplary MRI images of two patients and corresponding clinical data. Patient 1 shows a beginning cerebellar degeneration with only mild neurological symptoms; the NfL value is not elevated. Patient 2 illustrates advanced cerebellar atrophy with severe neurological deficit and significantly increased NfL.

## Discussion

A-T is a progressive and life-limiting disease associated with cerebellar ataxia due to progressive cerebellar degeneration. In addition to ataxia, which is described in detail, the presence of chorea, dystonia, oculomotor apraxia, athetosis, parkinsonism, and myoclonia are typical manifestations of the disease [32]. Especially in adult patients, a wide spectrum of movement disorders is reported, among which dystonia and chorea were the most frequent [33, 34]. While ataxia in the classic form of the disease is usually already apparent during the first year of life, the extrapyramidal symptoms mentioned above also play a substantial role in disease progression. They can lead to a higher degree of impairment and disability than the eponymous ataxia.

The neurological deterioration leads to a considerable limitation in everyday life. The children and adolescents are permanently dependent on the help of their guardians and are no longer independently able to perform even simple activities such as personal hygiene from adolescence onwards. Normally the progressive neurological deficit leads to permanent dependence on a wheelchair at a mean age of 8 years [35–37]. In addition to the psychosocial burden of permanent wheelchair dependence, this also creates a high medical burden through immobilization myopenia, osteopenia, and loss of lung capacity [38]. Dysphagia is an additional problem. Due to the uncoordinated swallowing, aspiration pneumonia is frequent and leads to a worsening of the lung disease [39], which is the leading cause of death in A-T patients with two-thirds of the cases [17].

During the first years of life, however, no cerebellar abnormalities are detectable in cerebral MRI scans [40], unless laborious voxel-based volumetry is performed, which may delay diagnosis and make it difficult to characterize the progression of cerebellar degeneration. During the course of the neurodegenerative disease, the cerebellum and the cerebrum are affected with lesions in the white matter and micro-infarctions [41]. Previous data showed that in older patients, extracerebellar MRI lesions were accompanied by deficiency of the GH/IGF-1 axis, malnutrition, and high ataxia scores [42]. Since imaging techniques require very laborious efforts to quantify the progression of disease, especially in its early stages, there is an urgent need to define easily obtainable, objective biomarkers of successive disease stages. Recently, the first quantitative MRI study in British A-T patients has been conducted [43].

Neurological scales such as SARA [24] and International Cooperative Ataxia Rating Scale (ICARS) [44] have been developed to monitor the course of ataxic movement disorders. For A-T, a special semi-quantitative score, the Neurological Examination Scale Toolkit (AT-NEST) [23, 45], was developed. These clinical scales require a high degree of patient cooperation and compliance, a difficulty especially in younger children. The scores thus have a limited value for identifying patients with rapid disease progression and a high risk of early wheelchair use at an early stage of the disease. Even in advanced stages of A-T, the scores lose their value, since the course of the disease for patients who are already wheelchair-bound becomes gradually more difficult to quantify by an increase in points.

In summary, this gives a devastating picture: there is a severe lack of screening tools that are appropriate for early assessment of a prognosis for A-T patients and for initiating corresponding therapies. Only suitable biomarkers will enable physicians to screen and treat severely affected patients before irreversible widespread neurological deficits have developed. While AFP is a well-established and mandatory biomarker for the diagnosis of A-T, because its elevation is a unique feature of the disease, it is not appropriate as a prognostic marker. In contrast, NfL is a tissue-specific biomarker for the prognosis and severity of neurodegeneration. Nevertheless, NfL is not disease-specific but elevated in numerous neurodegenerative processes and thus not appropriate for diagnosis. In keeping with this statement, the ROC curve (Fig. 5) clearly shows that AFP is the best marker to confirm the initial diagnosis and has excellent specificity and sensitivity. NfL is not adequate for primary diagnosis but has its value to map neurodegeneration. So far, only a few studies exist which used CSF to find potential biomarkers in A-T. Dzieciatkowska et al. characterized five potential protein dysregulations in CSF [46]. However, no children and adolescents were included; therefore, such biomarkers are unable to predict the development of A-T tissue breakdown. In 2018, the research team in Frankfurt demonstrated CSF ApoB excess and Reelin signaling deficits to reflect the neurodegeneration in A-T [47]. Another study of the German A-T cohort detected an altered albumin ratio and altered Reelin levels in CSF. The first finding would normally suggest a disturbed blood-brain barrier; however, no low-grade inflammation or altered cytokine levels were found compared to healthy controls, so albumin may be dysregulated because of osmotic stress, similar to AFP [48].

The present study measured serum NfL, which has already been shown to be a sensitive and specific marker of neurodegeneration in other diseases. We could show a significant increase of NfL when compared to healthy controls. The increase of NfL correlated significantly with age, increase of serum AFP, and SARA. Thus, we could show that NfL as a biomarker well documents the clinical progress of A-T. The physiological increase of NfL with age was 0.31 pg/mL/year in the healthy control group, about one-fifth below the increase of 1.49 pg/mL/year in the A-T cohort. Thus, although the two groups differed significantly in age, we were able to document a dramatically higher increase of this biomarker in the A-T cohort.

Elevated levels of NfL have been described in many neuroaxonal damage conditions such as HIV-associated dementia, amyotrophic lateral sclerosis (ALS), Creutzfeldt-Jakob disease, multiple sclerosis (MS), multi-system atrophy, cortico-basal degeneration, progressive supranuclear palsy, traumatic brain injury, fronto-temporal dementia, normal pressure hydrocephalus, dementia with Lewy bodies, Alzheimer dementia, and Parkinson’s disease associated with dementia [49].

Neurofilaments are part of the neural cytoskeleton and subject to constant renewal and degradation even in healthy subjects and are therefore released in small amounts into the CSF. By draining CSF through the lymphatic system, NfL enters the bloodstream where it can be easily extracted and determined [50]. While ELISA technique can quantify the phosphorylated form of neurofilament heavy chain (pNfH) only in CSF, it can measure NfL reliably also in serum. The more advanced single molecular array (SIMOA) technique is able to quantify both neurofilament components in serum [51]. However, it is currently unclear whether the increase of NfL in serum is caused by active remodeling in response to inflammation, hypoxia, infarction, or trauma or whether it is released passively during the neuronal remodeling and synaptic plasticity. In healthy controls, there is a physiological increase of neurofilaments with age in CSF and serum [27].

In multiple sclerosis, serum levels of NfL correlate with MRI findings, neurological examination, and disease activity [52]. A decrease in serum NfL levels with therapy response has also been shown for MS [52], so NfL may also become useful in A-T to demonstrate therapeutic efficacy once causal neuroprotective treatment is available.

In ALS, NfL and pNfH correlate with the extent of motor neuron degeneration [53]. NfL increases early in disease and remains on high concentrations [54]. Serum levels of NfL seem to correlate with the prognosis in ALS. Already in 1988, Monaco et al. published a manuscript on neuropathological abnormalities in one A-T patient, where Lewy bodies, cytoplasmic inclusions, and axonal spheroids in the brainstem nuclei and pathologies of the spinal cord similar to those of ALS were described [55]. In immunocytochemical studies, these protein aggregates were demonstrated to include neurofilament components [55].

Recently, it was also confirmed that in spinocerebellar ataxias, an increase in NfL precedes the onset of neurological symptoms. Thus, it has been shown that serum NfL elevation can be detected at the pre-ataxic stage of familial cerebellar syndromes, revealing earliest neuropathological changes to precede clinical manifestation [56]. In SCA3, levels of NfL correlated with disease severity [30].

In Friedreich’s ataxia, serum levels of NfL and pNfH were significantly elevated compared to healthy controls [51]. In curious contrast to all other neurodegenerative processes, in Friedreich’s ataxia, NfL serum levels seem to decrease with age, independent of clinical or genetic severity [57]. This is of particular interest, given that the current study demonstrates a significant increase of NfL with age in A-T.

There is an ongoing discussion whether serum NfL is particularly high in neurodegenerative disorders that affect the corticospinal pyramidal tract [58]. Apart from the cerebellar atrophy, the cerebral cortex is also involved as A-T progresses. This has been shown in various imaging studies, but so far it has seemed to play only a minor clinical role. To further clarify this pathomechanism, further imaging studies are needed, focusing on the relationship between brain lesions and their influence on NfL increase. British researchers recently reported that quantitative MRI demonstrates cerebellar abnormalities in A-T patients with negative linear correlation between age and fractional total cerebellar volumes compared to healthy controls [43]. Another possible explanation for the progressive increase is that NfL is also released from neurons in peripheral polyneuropathy as in Charcot-Marie-Tooth disease [59] and, in addition to the degenerative processes of the central nervous system, causes an increase in serum NfL in A-T in older patients. In line with this finding, we could show a significant correlation of NfL with peripheral neuropathy. Age may be a confounder in this context as neuropathy is an advanced clinical finding of A-T. We could also show a correlation with of NfL with myoclonus. Dystonia did not correlate with NfL. This may, however, be related to the small sample size (dystonia: n = 3 patients, myoclonus: n = 12 patients) and should be target of further research.

There was a significant correlation of NfL with diabetes and hepatopathy, suggesting a parallel progression of neurodegeneration and metabolic risk factor. Summarized, NfL, neuropathy, diabetes and hepatopathy show rapid progression with age and disease duration.

NfL is a promising biomarker whose relevance should be the focus of further studies. In particular, it could be used to detect patients with severe disease progression at an early stage and to introduce one of the new treatment options before reaching a severe neurological disability. There are currently no such screening tools as MRI and neurological scales only document irreversible loss of function. The central aim of further research should now be the validation of NfL as a screening tool that allows patients to be identified and receive potent treatments before a relevant loss of cerebellum and motor skills. The collection of longitudinal data and the validation of NfL in upcoming clinical trials should be the objective of further research projects.

To our best knowledge, this is the first scientific report about increased levels of NfL in A-T. In addition to that, we could demonstrate a progression with age and neurological deficit. However, this study has some limitations. The study does not include longitudinal data. In addition, we have only few corresponding MRI images at the time of NfL measurement and neurological examination to support our results morphologically. Rather, we understand the presented work as a pilot project that shows for the first time that NfL is a potential biomarker of neurodegeneration and disease progression in A-T. However, it cannot be used for diagnosis, as it is not disease-specific. Another weakness is that due to the mean age, not all patients were compliant or cooperative enough to perform neurological scoring. To ameliorate compliance, shorter examination duration, and because of a higher interrater reliability in this mainly pediatric cohort, we used the SARA score only. Unfortunately, we do not have data on the duration of the disease to determine an exact symptom onset for all patients. The study included only classical patients with early disease onset. Besides the aging effect, the influence of mutation severity and personal and genetic resilience is certainly crucial for the individual course of neurodegeneration, which we want to map with NfL. For this, further research with larger cohorts is needed, also in the context of future pharmacological studies.

## Conclusion

NfL is a promising biomarker for neurodegeneration in A-T that correlates with age and neurological deficit. Further research and longitudinal data are needed to evaluate its relevance in predicting disease course and therapy response.

## Data Availability

The datasets used and/or analyzed during the current study are available from the corresponding author on reasonable request.

## Abbreviations

AFP: alpha fetoprotein
ALS: amyotrophic lateral sclerosis
A-T: ataxia telangiectasia
AT-NEST: Neurological Examination Scale Toolkit
CSF: cerebrospinal fluid
ICARS: International Cooperative Ataxia Rating Scale
MRI: magnetic resonance imaging
MS: multiple sclerosis
NfL: neurofilament light chain
pNfH: neurofilament heavy chain
SARA: scale for the assessment and rating of ataxia
SCA3: spinocerebellar ataxia type 3
SDs: standard deviations
SIMOA: single molecular array
WHO: World Health Organization
